# Supplementary material for: Peripheral Myelin Protein 22 Gene Mutations in Charcot-Marie-Tooth Disease Type 1E Patients
Source: Genes (Basel). 2022 Jul 8;13(7):1219. doi: 10.3390/genes13071219 (PMC9321036; doi:10.3390/genes13071219)
Supplement: Supplementary file 1 [file genes-13-01219-s001.zip › Suppl Table S1.pdf]

Table S1. In silico prediction of *PMP22* mutations.

Mutations[1]		dbSNP accession No	GERP	In silico *a*nalyses[2]		
**Nucleotide**	**Amino acid**			**PRO**	**PP2**	**MU**
c.35A>G	p.H12R	rs1909248652	3.37	-7.52*	0.00	0.66
c.47T>G	p.L16R	NR	3.36	-3.46*	0.76*	-0.38*
c.68C>G	p.T23R	rs906563423	3.58	-5.61*	1.00*	0.84
c.179-1G>A	p.?	rs1597608220	5.61	-	-	-
c.215C>T	p.S72L	rs104894621	5.61	-4.85*	1.00*	0.13
c.245T>C	p.L82P	rs878853113	4.52	-3.98*	1.00*	-1.00*
c.256C>T	p.Q86X	rs11545341	5.61	-	-	-
c.281delG	p.G94Afs*16	NR	5.76	-	-	-
c.280_281delinsT	p.G94Sfs*16	rs864622678	-0.53/5.76	-	-	-
c.298G>A	p.G100R	rs1597607651	5.76	-5.14*	1.00*	0.15
c.318delT	p.G107Vfs*3	NR	-0.563	-	-	-
c.319+1G>T	p.?	NR	5.76	-	-	-
c.323T>C	p.L108P	NR	5.16	-5.93*	1.00*	-1.00*
c.325T>C	p.C109R	rs1597597876	5.16	-7.33*	1.00*	0.44

[1] Reference nucleotide and amino acidn sequences: NM_000304.4 and NP_000295.1.

[2] In silico scores of PROVEAN (PRO) < −2.5, PolyPhen-2 (PP2) ~1, and MUpro (MU) < 0 indicate pathogenic prediction (* denotes a pathogenic prediction).

Abbreviations: CMT1E: Charcot-Marie-Tooth disease type 1E, GERP: genomic evolutionary rate profiling.
