# Supplementary material for: Peripheral Myelin Protein 22 Gene Mutations in Charcot-Marie-Tooth Disease Type 1E Patients
Source: Genes (Basel). 2022 Jul 8;13(7):1219. doi: 10.3390/genes13071219 (PMC9321036; doi:10.3390/genes13071219)
Supplement: Supplementary file 1 [file genes-13-01219-s001.zip › Suppl Table S2.pdf]

Table S2. Clinical features of Charcot-Marie-Tooth disease type 1E patients with mutations in *PMP22* gene.

Patient ID	Sex	Ages (yr)		CMT	CMT	CMT	CMT	FDS	MRC[1]		Sensory	DTR[2]	Hearing	Foot
		Exam	Onset	NSv2	NS-R	ES	ES-R		UEx	LEx	loss	Knee/ankle	loss	deformity
FC618 (II-1)	M	22	1	31	38	23	29	7	+++	+++	Yes	A/A	No	Yes
FC541	M	13	12	ND	ND	ND	ND	1	+	++	Yes	A/A	Yes	Yes
FC50 (II-2)	F	84	31	24	28	20	24	4	++	++	Yes	A/A	Yes	Yes
FC50 (III-7)	F	64	25	20	23	16	19	4	++	+++	Yes	A/A	Yes	Yes
FC50 (III-8)	M	49	19	23	28	18	22	3	++	++	Yes	A/A	Yes	Yes
FC50 (III-11)	F	45	26	21	24	17	20	4	++	+++	Yes	A/A	Yes	Yes
FC50 (IV-8)	M	20	12	15	17	11	13	1	+	++	Yes	A/A	Yes	Yes
FC50 (IV-11)	M	15	11	15	17	11	13	2	+	++	Yes	A/A	Yes	Yes
FC303	M	20	10	13	16	9	12	1	+	+	Yes	D/A	No	Yes
FC680 (I-1)	M	70	27	21	29	17	22	3	++	++	Yes	A/A	Yes	Yes
FC680 (II-1)	M	34	9	20	23	16	19	2	+	+	Yes	A/A	Yes	Yes
FC970 (II-3)	F	40	29	9	10	9	10	1	+	+	Yes	D/D	No	Yes
FC970 (II-4)	F	39	34	14	16	11	13	1	+	+	Yes	D/D	Yes	No
FC970 (III-1)	F	13	12	7	11	6	9	0	-	+	No	N/D	No	No
FC285 (II-1)	M	16	<1	31	38	23	29	7	+++	+++	Yes	A/A	No	Yes
FC376 (II-1)	F	11	2	32	38	24	29	7	+++	+++	Yes	A/A	No	Yes
FC732 (II-2)	F	7	2	29	35	21	26	7	++	+++	Yes	A/A	No	Yes
FC829 (III-3)	F	12	<1	25	29	17	10	7	++	+++	Yes	A/A	No	Yes
FC895	M	7	<1	31	35	23	26	6	+++	+++	Yes	A/A	No	No
FC416 (II-2)	F	28	<1	30	35	22	26	7	++	+++	Yes	A/A	No	Yes
FC1102 (I-2)	F	47	16	ND	ND	ND	ND	2	+	++	Yes	D/A	No	Yes
FC1102 (II-1)	M	21	14	15	17	11	13	2	+	++	Yes	D/A	No	Yes
FC1325 (II-3)	M	54	8	13	20	15	23	3	+	++	Yes	A/A	Yes	Yes
FC1325 (III-1)	M	31	20	8	12	11	15	2	+	+	Yes	D/A	No	Yes
FC1061 (III-1)	F	26	<1	31	37	23	28	7	+++	+++	Yes	A/A	No	Yes
FC1088	F	48	5	30	37	22	28	7	+++	+++	Yes	A/A	No	Yes
FC1140 (II-2)	F	6	1	22	29	16	20	4	++	+++	Yes	A/A	No	No
FC35 (II-5)	M	34	20	ND	ND	ND	ND	3	+	++	Yes	A/A	Yes	Yes
FC35 (II-6)	M	32	19	15	22	12	17	3	+	++	Yes	A/A	Yes	Yes

FC608	M	39	31	19	25	12	17	2	+	++	Yes	A/A	No	Yes
FC1060	M	33	21	15	23	9	12	3	+	++	Yes	D/A	No	No
FC284 (II-1)	F	15	<1	34	37	26	28	7	+++	+++	Yes	A/A	No	Yes

[1] Muscle weakness in upper limbs (UEx): -: normal, +: intrinsic hand weakness of 4/5 on the Medical Research Council (MRC) scale, ++: intrinsic hand weakness < 4/5 on the MRC scale, +++: proximal weakness. Muscle weakness in lower limbs (LEx): +: ankle dorsiflexion of 4/5 on the MRC scale, ++: ankle dorsiflexion < 4/5, +++: proximal weakness.

[2] Deep tandon reflex (DTR): D: diminished, A: absent, N: normal reflex.

Abbreviations: A: absent action potential, CMAP: compound muscle action potential, CMTNSv2: CMT neuropathy score ver. 2, CMTNS-R: Rasch-modified CMTNS, CMTES: CMT examination score, CMTES-R: Rasch-modified CMTES, ND: not determined, MNCV: median nerve conduction velocity, ND: not determined, SNAP: sensory nerve action potential, SNCV: sensory nerve conduction velocity.
