# Supplementary material for: Peripheral Myelin Protein 22 Gene Mutations in Charcot-Marie-Tooth Disease Type 1E Patients
Source: Genes (Basel). 2022 Jul 8;13(7):1219. doi: 10.3390/genes13071219 (PMC9321036; doi:10.3390/genes13071219)
Supplement: Supplementary file 1 [file genes-13-01219-s001.zip › Suppl Table S3.pdf]

Table S3. Electrophysiological values of Charcot-Marie-Tooth disease type 1E patients with mutations in *PMP22* gene.

Patient	Age at exam (yrs)	Motor nerve conduction studies												Sensory nerve conduction studies					
		Median nerve			Ulnar nerve			Peroneal nerve			Tibial nerve			Median nerve		Ulnar nerve		Sural nerve	
		DTL	CMAP	MNCV	DTL	CMAP	MNCV	DTL	CMAP	MNCV	DTL	CMAP	MNCV	SNAP	SNCV	SNAP	SNCV	SNAP	SNCV
FC618 (II-1)	22	A	A	A	A	A	A	A	A	A	A	A	A	A	A	A	A	A	A
FC50 (III-7)	64	14.2	0.7	14.2	5.0	11.0	18.6	A	A	A	8.7	0.6	12.4	A	A	3.1	18.6	A	A
FC50 (III-11)	45	9.6	4.1	14.3	6.0	6.8	16.0	A	A	A	A	A	A	A	A	A	A	A	A
FC50 (IV-11)	18	7.9	8.9	21.4	5.9	11.7	22	10.1	3.5	19.1	11.8	3.4	18	A	A	A	A	A	A
FC50 (II-2)	84	A	A	A	5.0	8.2	18.1	A	A	A	A	A	A	A	A	A	A	A	A
FC50 (IV-8)	20	6.7	6.1	20.7	5.6	11.4	20.6	14.6	0.3	19.1	7.8	7.1	13.9	A	A	A	A	A	A
FC50 (III-8)	49	9.4	1.2	17.7	5.6	4.3	20.8	8.5	0.6	15.2	11.7	0.9	14.6	A	A	A	A	A	A
FC303	20	6.2	11.6	24	5.4	11.1	20.9	8.4	5	20.6	8	6.6	18.1	A	A	A	A	A	A
FC680 (I-1)	70	8.9	8.1	18.8	5.6	5.2	19.2	A	A	A	A	A	A	2.4	18.2	4.5	17.5	A	A
FC680 (II-1)	34	7.9	3.3	17.7	5.0	6.5	15.7	A	A	A	A	A	A	A	A	A	A	A	A
FC970 (III-1)	13	5.2	17.4	35.5	3.5	13	32.7	8.7	0.9	28.8	6.2	5.4	24.4	20.9	29.2	14.4	28.9	10	24.6
FC970 (II-3)	40	4.4	19.0	46.8	2.8	14.5	42.3	6.3	7.4	34.5	5.5	6.8	32.4	28.6	35.3	15.5	36.6	1.2	26.4
FC970 (II-4)	39	5.3	12.3	29.9	3.6	11.2	24.0	7.4	5.5	21.6	8.5	0.9	25.2	9.4	26.2	5.3	26.5	A	A
FC285 (II-1)	16	A	A	A	A	A	A	A	A	A	A	A	A	A	A	A	A	A	A
FC376 (II-1)	11	A	A	A	A	A	A	A	A	A	A	A	A	A	A	A	A	A	A
FC732 (II-2)	7	A	A	A	A	A	A	A	A	A	A	A	A	A	A	A	A	A	A
FC829 (III-3)	7	A	A	A	A	A	A	A	A	A	A	A	A	1.8	20.8	1.0	21.6	A	A
FC895	7	A	A	A	A	A	A	A	A	A	A	A	A	A	A	A	A	A	A
FC416 (II-2)	24	A	A	A	A	A	A	A	A	A	A	A	A	A	A	A	A	A	A
FC1102 (II-1)	21	6.8	6.3	13.6	4.9	8.3	14.7	A	A	A	11.2	1.3	14.6	A	A	A	A	A	A
FC1325 (II-3)	54	12.1	1.8	11.4	7.5	1.5	11.8	A	A	A	A	A	A	A	A	A	A	A	A
FC1325 (III-1)	31	9.2	6.8	12.5	6.4	9.5	13.3	13.7	0.7	8.8	13.1	1.3	8.7	A	A	A	A	A	A
FC1061 (III-1)	26	A	A	A	A	A	A	A	A	A	A	A	A	A	A	A	A	A	A
FC1088	48	A	A	A	A	A	A	A	A	A	A	A	A	A	A	A	A	A	A
FC1140 (II-2)	6	A	A	A	A	A	A	A	A	A	A	A	A	A	A	A	A	A	A
FC35 (II-6)	32	6.3	10.0	29.5	6.1	2.7	28.7	A	A	A	A	A	A	A	A	A	A	A	A
FC608	39	9.1	0.5	6.0	8.2	0.5	10.7	A	A	A	A	A	A	A	A	A	A	A	A

FC1060	33	A	A	A	12.1	3.2	10.0	A	A	A	A	A	A	A	A	A	A	A	A
FC284 (II-1)	15	A	A	A	A	A	A	A	A	A	A	A	A	A	A	A	A	A	A

Normal DTL values: motor median nerve: <3.6 ms, ulnar nerve: <2.5 ms, peroneal nerve: <4.8 ms, and tibial nerve: <5.1 ms. Normal NCV values: motor median nerve: ≥50.5 m/s, ulnar nerve: ≥51.1 m/s, peroneal nerve: ≥41.2 m/s, tibial nerve: ≥41.1 m/s, sensory median nerve: ≥39.3 m/s, ulnar nerve: ≥37.5 m/s, and sural nerve: ≥32.1 m/s. Normal amplitude values: motor median nerve: ≥6 mV, ulnar nerve: ≥8 mV: peroneal nerve: ≥1.6 mV, tibial nerve: ≥6 mV, sensory median nerve: ≥8.8 μV, ulnar nerve, ≥7.9 μV; sural nerve, ≥6.0 μV.

Abbreviations: A: absent action potential, CMAP: compound muscle action potential, CMT1E, CMT type 1E, DTL: distal terminal latency, SNAP: sensory nerve action potential, SNCV: sensory nerve conduction velocity.
